# Seroprevalence of Selected Tick Borne Pathogens and Diversity and Abundance of Ixodid Ticks (Acari: *Ixodidae*) at the Wildlife-Livestock Interface in Northern Botswana

**DOI:** 10.3389/fvets.2020.00187

**Published:** 2020-05-05

**Authors:** Obuile O. Raboloko, Solomon S. Ramabu, Laure Guerrini, Ferran Jori

**Affiliations:** ^1^Veterinary Sciences, Botswana University of Agriculture and Natural Resources, Gaborone, Botswana; ^2^Department of Veterinary Services, Ministry of Agriculture, Gaborone, Botswana; ^3^Paul G. Allen School for Global Animal Health, Washington State University, Pullman, WA, United States; ^4^UMR Animal, Health, Territories, Risks and Ecosystems (ASTRE), CIRAD-INRA- Uni. Montpellier, Campus International de Baillarguet, Montpellier, France; ^5^CIRAD, UMR ASTRE, RP-PCP, Harare, Zimbabwe

**Keywords:** tick borne diseases, *Anaplasma* spp., *Babesia* spp., *T*. *parva*, seroprevalence, ticks, veterinary fence, Botswana

## Abstract

Ticks and tick borne diseases (TBDs) undermine livestock production with considerable economic losses to livestock producers in endemic areas worldwide. Despite the impact of ticks and TBDs in livestock production, there is a paucity of information on ticks and diseases they transmit in Botswana. To address this gap, a cross-sectional study was conducted to determine (i) the seroprevalence of selected tick borne (TB) pathogens and (ii) the diversity and abundance of ixodid ticks among 301 cattle foraging around two protected areas in northern Botswana, differing by the presence or absence of a physical barrier (fence) separating wildlife and livestock. Competitive inhibition enzyme linked immuno-sorbent assay (cELISA) was used to test for *Anaplasma* spp. infection and Indirect Fluorescence Antibody Test (IFAT) was used to test for *Theileria parva, Babesia bovis*, and *B*. *bigemina*. Ticks were identified morphologically at either genus or species level. Seroprevalence of cattle was found to be 90% for *Anaplasma* spp., followed by 38.6% for *Babesia* spp. and 2.4% for *T*. *parva*. Except for *Babesia* spp., comparisons of the seroprevalence of the selected haemoparasites between the two wildlife-livestock interface areas were not significantly different. The overall prevalence of ticks was found to be 73.4% with *Amblyomma variegatum* being the most abundant (53.1%) followed by *Rhipicephalus evertsi evertsi* (31.7%) and *R*. (*B*.) *decoloratus* (7.7%). Except for *Babesia* spp., comparisons of the seroprevalence of the selected haemoparasites between the two study areas were not significantly different while comparisons of the burden of tick infestation between the study sites revealed significant difference for *A*. *variegatum* and *R*. *evertsi evertsi* with both tick infestations higher where there is no barrier. Our work provided baseline data on TBD pathogens and tick infestation in cattle populations exposed to different levels of contact with adjacent buffalo populations. The presence of a veterinary fence did not significantly influence the seroprevalence of the selected TBD pathogens (except for *Babesia* spp.) but seemed to reduce tick burdens in cattle. Findings from this study can be used for guiding future epidemiological study designs to improve our understanding of ticks and TBDs dynamics in northern Botswana.

## Introduction

Ticks (Acari: *Ixodidae*) and tick borne haemoparasites including anaplasmosis, babesiosis, ehrlichiosis (cowdriosis), and theileriosis limit livestock production in endemic areas worldwide including sub-Saharan Africa (1, 2). Ticks and TBDs affect nearly 80% of the world's cattle population, with estimated annual global costs ranging from US$ 14-19 billion (3). Limited information exists on the impact of TBDs in the national economies of sub Saharan countries. In Tanzania, for example, the annual losses due to TBDs in the livestock sector were estimated at US$ 364 million. An estimated 70% of these losses were theileriosis, the remaining 30% being represented by anaplasmosis, babesiosis, and cowdriosis (4). In South Africa, annual losses due to TBDs are estimated to range between R70 and R550 million (5). Taken together, this may lead to increased poverty and impact negatively on food security. The level of interaction between wild and domestic animals relies on host behavior, which is influenced by environmental conditions and land use (protected areas or communal land, veterinary fences and infrastructure (6, 7). The coexistence of wildlife, livestock and humans at different wildlife-livestock interfaces, may facilitate pathogen transmission affecting animal, wildlife and public health (8). Kock (9) reported that wildlife can contribute to livestock TBDs by serving as both sources and maintenance hosts for pathogens causing diseases. The African buffalo (*Syncerus caffer*), for example, is the natural reservoir host of *Theileria parva* which causes East Coast fever (ECF) and Corridor Disease (10). Other TB pathogens of veterinary importance such as *Anaplasma marginale, Ehrlichia ruminantium, B. bigemina*, and *B*. *bovis* causing Anaplasmosis, heartwater, and babesiosis) have also been reported in African buffalo (11, 12). In Botswana, a recent study by Eygelaar et al. (2), revealed the presence *Theileria* spp. and *Anaplasma* spp. in buffalo in two protected areas in northern Botswana. The buffalo does not show clinical disease following infection with TBDs but as a reservoir host, it can transmit TBDs pathogens to cattle when both species share infected ticks during their interaction resulting in decreased animal production (2, 8). On the other hand, it has been reported that domestic livestock play a role in facilitating the spread of tick borne haemoparasites among the wild population (13). Exploring the prevalence of tick borne pathogens and tick infestation in cattle at the edge of protected areas in Southern Africa including Botswana, where interactions between domestic and wild ruminants are common and widespread is necessary not only for herd health but also for wildlife conservation and public health. In the case of zoonosis, the health of rural people with limited access to health services can suffer from the spill over of pathogens from domestic and wildlife populations (7). Little is known on TBDs pathogens in cattle at the wildlife-livestock interface in northern Botswana. Therefore, this study aimed to determine the seroprevalence of selected TBDs pathogens (*Anaplasma* spp., *B*. *bigemina, B*. *bovis*, and *T*. *parva*) and to document diversity, abundance, and spatial distribution of ixodid tick species circulating in cattle at two different wildlife-livestock interface areas in northern Botswana.

## Materials and Methods

### Study Sites

**Figure 1:**
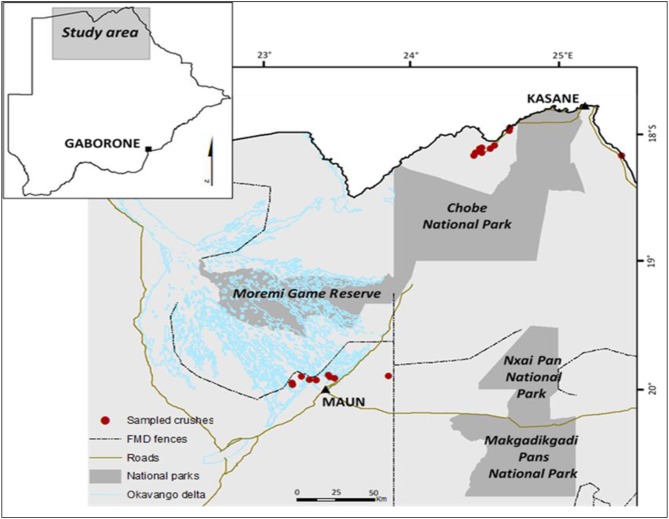
Map showing location of sampling sites (Maun west and Chobe west) in northern Botswana.

The study was conducted between April and August 2015 in northern Botswana, Maun west (MW) located in Maun district, and Chobe west (CW) located in Kasane district. Both districts encompass iconic protected areas such as the Okavango Delta (OD) in Maun district and Chobe National Park (CNP) in Kasane which are the largest wildlife areas and tourism attractions of the country and part of the Kavango-Zambezi Trans-frontier Conservation Area (KAZA TFCA). IN CW, the Chobe river forms the border between Botswana and Namibia and there is no veterinary fence (no fence interface) separating wildlife from livestock populations, while in MW there is a fence surrounding the edge of the OD (fenced interface) (Figure 1). In Southern Africa, veterinary cordon fences are prone to be damaged by elephants and floods leading to buffalo incursions into livestock areas or visa-visa (14, 15). Chobe river and the Okavango delta are the main sources of water for livestock and wildlife in the study sites providing abundant water throughout the year. As in other areas of Botswana MW and CW are characterized by three seasons; namely: the wet season (November–March), the cool dry season (April–July), and the hot dry season (August–October). This seasonal pattern influences the movement and contact of livestock and wildlife with the peak interaction during the dry season as compared to the wet season. Vegetation is predominantly deciduous dry woodland and scattered grassland (2). The total cattle census in northern Botswana is estimated at 210,000 heads, with 3,500 in MW and 4,530 in CW and (Department of Veterinary Services, Botswana, unpublished Records, 2015). In both study areas, cattle are raised under traditional (communal) system characterized by shared grazing.

Using a global positioning system (GPS) device (Garmin eTrex® Legend C), geographical coordinates of each sampled localities (crush pens) were taken. In MW, the crushes were selected within 10 km from the veterinary cordon fence while in CW, within 10 km from Chobe river (Figure 1). In both areas, cattle of different age groups (calves and adults) based on dentition, both sexes (male and females), and breeds comprising of indigenous Tswana (*B*. *indicus*) and other breeds (produced by crossing of Tswana breed with imported breeds), presented to the crush pens were selected for sampling in this study. Sampling in this study was done in winter and in two parts: seroprevalence in April (dry cool season) and tick collection in August (hot dry season). The same sample size was implemented for seroprevalence and tick sampling but in different animals.

### Tick Sampling and Identification

A total of 301 cattle were sampled. Ticks were detached by manual (hand) removal holding the tick at the basis of the capitulum and gently removing it by exerting a horizontal pull to the body surface so as not to lose the mouthpart. During sampling, an effort was made to collect from different body parts (head, ears, neck, dewlap, legs, perineum, belly, udder, scrotum, and the base of the tail) in order to obtain a representative idea of all the species present using tick collection method described by Londt et al. (16). Specimens were preserved in 70% ethanol. The sample vials were labeled indicating owner's name, crush pen, date of collection and animal information (age, sex, and breed), and month of collection. Ticks were identified and categorized to genus and species levels using a stereoscope (80-fold magnification) and following morphological descriptions made by Walker et al. (17). The geographic coordinates of each locality was recorded and used for the production of maps of the distribution of the tick species in Arcgis 10.4.

### Sampling and Sample Size Determination

The prevalence of antibodies of *Anaplasma* spp., *Babesia* spp., and *Theileria parva* in cattle in Botswana, was unknown therefore prevalence from previous studies in neighboring countries were used to determine sample size. Thus, the expected prevalence rate of 26% for *A*. *marginale* (18) in South Africa using cELISA with specificity of 99.5%, sensitivity of 98% (19), a 95% level of confidence and a 10% tolerable error was used to predict prevalence of antibodies of tick borne pathogens. A software Free calc® Version 2—Survey tool box described by Humphry et al. (20) was used to calculate the sample size. The sample size to detect antibodies against anaplasmosis was estimated at a minimum of 296 cattle. However, a total of 301 cattle were sampled. The sample size was distributed proportionally to the crushes according to the available census data from the veterinary services. Within every herd, a systematic random sampling was applied.

### Serological Testing

At each epidemiological unit represented by a crush, the cattle to be sampled were systematically randomly selected with sampling interval of 10 animals. Cattle were manually restrained and about 4 ml of blood was obtained by venepuncture of the jugular or coccygeal veins of each animal using plain vacutainer tubes. The blood was then centrifuged and sera harvested into cryovials, labeled and stored frozen at −20°C until the samples were tested.

### cELISA Test for *Anaplasma* spp.

Antibodies against *Anaplasma* spp. in serum were detected by MSP-5 competitive enzyme linked immunosorbent assay (cELISA) using commercially available *Anaplasma* Antibody Test Kit, cELISA (VMRD Inc., Pullman, WA, USA) [sensitivity (se) 95%; specificity (sp) 98%]. The test was performed according to the manufacturer's instruction at Botswana University of Agriculture and Natural Resources (BUAN), Botswana. Briefly, 96-well flat-bottom plates coated with recombinant MSP- 5 were incubated with the sample sera for 1 h at room temperature (21–25°C) and washed 2 times with a wash solution. Then, antibody peroxidase conjugate was added to each well and incubated for an additional 20 min at room temperature. After incubation, plates were washed four times and *o*-phenylenediamine dihydrochloride was added to the plates and incubated for an additional 20 min. A stop solution was added before the plate was read. An ELISA reader (Multiskan FC, Thermo Scientific, and Waltham, USA) was used to measure the optical density at 620 nm wavelength. A cut off of 30% inhibition was used to differentiate between positive and negative samples. Serum samples with ≥30% inhibition were considered positive while samples with <30% inhibition were considered negative. The percent inhibition (% I) was calculated using formula: 100 – [(Test sample optical density/Mean negative control sera optical density) × 100]

### IFAT for *T. parva, B. bovis*, and *B. bigemina*

The sampling process allowed obtaining sera from 301 cattle. Unfortunately, six samples were lost making 295 serum samples available for testing. As the samples were coming from FMD zones, prior to testing, sera was subjected to heat treatment at 60°C during 30 min. This procedure was reported not to affect the test performance. The schizont antigen IFAT (se of 96% and sp of 95%) was carried out according to the method previously described by Burridge and Kimber and Goddeeris et al. (21–23) with slight changes at Agricultural Research council Onderstpoort Veterinary Research Council Institute (ARC-OVI), Republic of South Africa. Phosphate-buffered saline solution (PBS) was used to make 2-fold dilutions of the test and control sera of 1/80, 1/160 in 96 well micro-liter plates. Diluted test serum was incubated at 37°C for 60 min with the antigen fixed on the glass slides. Unbound antibodies are removed by washing them off. Bound antibodies are revealed by incubating the antigen-antibody complexes (60 min) with anti-species (e.g., anti-bovine) Immunoglobulin G (IgG) antibodies conjugated to a fluorescence compound—fluorescein isothiocyanate (FITC). Unbound FITC-conjugate is washed away leaving only the FITC-conjugate bound to test serum antibody-antigen complex. The fluorescing complexes (for a positive reaction) are read with a fluorescence microscope at 50x and 100x objectives. No fluorescing complexes are observed in a negative reaction and dull non-specific fluorescence may be observed. A titer of ≥1:80 were considered as positive reactions (Olivier Matthee, personal communication).

### Statistical Analysis

Data from serological tests was entered into Microsoft excel spread sheet, 2007. Seroprevalence was determined by expressing positive sera as a percentage of the total number of sera tested while the prevalence of ticks was determined by dividing the number of animals infested by the sample size expressed as a percentage. Risk factors potentially affecting seroprevalence and tick infestation such as area, age, sex and breed were considered in this analysis. Specifically, the chi-square test calculation was used to determine the association of the risk factors and selected tick borne pathogen seroprevalence and tick infestation using SAS (24). *P* < 0.05 were considered statistically significant.

## Results

### Tick Infestation and Distribution

**Figure 2:**
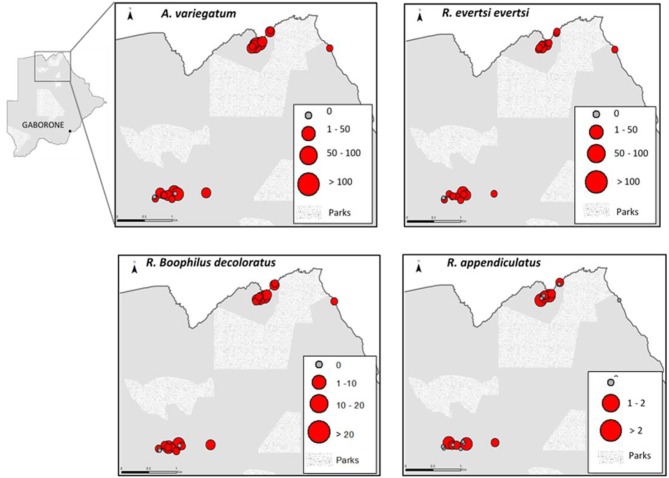
Spatial distribution of tick species at the wildlife-livestock interface in northern Botswana (Maun west and Chobe west).

The overall prevalence of ticks on cattle, without taxonomic and area differentiation was 73.4% (221/301) CI 95% [68.1–78.1]. A total of 2,522 ixodid ticks (1,415 males and 1,107 females) were collected from cattle at the two study sites. Three tick genera *Amblyomma, Rhipicephalus* (including *Boophilus* sub-genus), and *Hyalomma* and seven species were identified: *A. variegatum, R. evertsi*-*evertsi, R*. (*B.) decoloratus, R. appendiculatus, H. marginatum rufipes, H. truncatum*, and *R. sanguineous*. *Amblyomma*, was the most abundant and widely distributed genus in all study sites 53.1% (1,338/2,522) CI 95% [51.1–55.0] followed by *Rhipicephalus*, 40.8% (1,030/2,522) CI 95% [38.9–42.7] and *Hyalomma*, 6.1% (154/2,522) CI 95% [5.2–7.1]. *A*. *variegatum* was the most prevalent and widely distributed species (53.1%) followed by *R. evertsi evertsi* (31.7%); *R. (B.) decoloratus* (7.7%); *H. marginatum rufipes* (4%); *H. truncatum* (2.1%) *R. appendiculatus* (1%), respectively (Table 1, Figure 2).

**Table 1:** A comparison of ixodid tick species prevalence (%) per area, age, sex, and breed at the wildlife-livestock interface areas in northern Botswana (Maun west and Chobe west).

**Variables**		***A. variegatum***	***R. evertsi evertsi***	***R. (B.) decoloratus***	***R. marginatum rufipes***	***R. truncatum***	***R. appendiculatus***	***R. sanguineous***
Area	Maun west^a^	**79/151 (52.3%)**	**57/151 (37.7%)**	39/151 (25.8%)^a^	5/151 (3.3%)	24/151 (15.9%)	9/151 (6.0%)	1/151 (0.7%)
	Chobe west^b^	**106/150 (70.6%)**	**78/150 (52%)**	57/150 (38%)^a^	26/150 (17.3%)	14/150 (9.3%)	11/15 (73.3%)	1/151 (0.7%)
Age	Adults	**170/256 (66.4%)**	118/256 (46.1%)	**79/256 (30.9%)**	38/256 (14.8%)	**38/256 (14.8%)**	19/256 (7.4%)	2/256 (0.8%)
	Calves	**15/45 (33.3%)**	17/45 (37.7%)^a^	**7/45 (15.6%)**	3/45 (6.7%)	**0/45 (0.0%)**	1/45 (2.2%)	0/45 (0.0%)
Sex	Female	**149/224 (66.5%)**	**109/224 (48.7%)**	**71/224 (31.7%)**	34/224 (15.2%)	30/224 (13.4%)	15/224 (6.7%)	1/224 (0.4%)
	Male	**36/77 (46.8%)**	**26/77 (33.8%)**	**15/77 (19.5%)**	7/77 (9.1%)	8/77 (10.4%)	5/77 (6.5%)	1/77 (1.3%)
Breed	Tswana	**179/285 (62.8%)**	130/285 (45.6)	83/285 (29.1%)	38/285 (13.3%)	34/285 (11.9%)	19/285 (6.7%)	2/285 (0.7%)
	Other breeds	**6/16 (37.5%)**	5/16 (31.3%)	3/16 (18.8%)	3/16 (18.8%)	4/16 (25%)	1/16 (6.25%)	0/16 (0.0%)

a *A fence barrier separates livestock from wildlife (fenced interface)*.

b *There is no barrier separating livestock from wildlife (no fence interface)*.

*Significant differences (p ≤ 0.05) between variables are boldfaced*.

### A Comparison Between Areas

The comparison of *A. variegatum and R. evertsi evertsi* between study zones indicated that the infestation with these tick species were significantly higher (*p* = 0.001) and (*p* = 0.01), respectively in Chobe west where there is no fence between livestock and wildlife compared to Maun west where a fence is present (Table 1).

### Influence of Age, Sex, and Breed

There was a significant difference (*p* ≤ 0.05) between age of cattle that were infested with *A*. *variegatum, R*. (*B*.) *decoloratus*, and *H*. *truncatum* with adults having a higher infestation than calves. There was also significant difference (*p* ≤ 0.05) between sex of the animals that were infested with *A*. *variegatum, R*. *evertsi evertsi*, and *R* (*B*.) *decoloratus* with females being more prone to infestation than males. Except for *A*. *variegatum*, which was more prevalent in Tswana compared to other breeds, there was no significant difference in infestation by ticks between breeds (Table 1).

### Seroprevalence of Selected TBD Pathogens in Cattle in Northern Botswana

The seroprevalence of selected TBDs in Maun west (fenced interface) and Chobe west (unfenced interface), was determined using serological techniques. Seroprevalence of cattle in the two areas combined was found to be 90, 95% CI [86.1–92.9] for *Anaplasma* spp. being the highest, followed by *Babesia* spp. at 38.6, 95% CI [33.27–44.31] and the lowest was *T. parva* only at 2.4, 95% CI [1.15–4.82]. The test for *Babesia* spp. delineated the organisms into *Babesia bovis* and *Babesia bigemina*. *B. bigemina* had a higher prevalence (29.5%) compared to *B. bovis* (9.2%). Observed seroprevalence for the TBDs is given in (Table 2).

**Table 2:** A comparison of selected TBD pathogens seroprevalence (%) per area, age, sex, and breed at wildlife-livestock interface areas in northern Botswana (Maun west and Chobe west).

**Variables**		***Anaplasma* spp**.	***B. bovis***	***B. bigemina***	***T. parva***
Area	Maun west^a^	132/151 (87.5%)	**21/145 (14.5%)**	**22/145 (15.2%)**	5/145 (3.4%)
	Chobe west^b^	139/150 (92.6%)	**66/150 (44%)**	**5/150 (3.3%)**	2/150 (1.3%)
Age	Adults	**237/256 (92.6%)**	**71/251 (28.3%)**	25/251 (10%)	7/251 (2.8%)
	Calves	**34/45 (75.6%)**	**16/44 (36.4%)**	2/44 (4.2%)	0/44 (0%)
Sex	Male	78/86 (90.7%)	32/76 (42.1%)	2/76 (2.6%)	1/76 (1.3%)
	Female	193/251 (89.8%)	55/219 (25.1%)	25/219 (11.4%)	6/219 (2.7%)
Breed	Tswana	260/285 (90.9%)	84/281 (29.9%)	27/281 (9.6%)	7/281 (2.5%)
	Other breeds	11/15 (73.3%)	3/14 (21.4%)	0/14 (0%)	0/14 (0%)

a *A fence barrier separates livestock from wildlife (fenced interface)*.

b *There is no barrier separating livestock from wildlife (no fence interface)*.

*Significant differences (p ≤ 0.05) between variables are boldfaced*.

### A Comparison Between Areas

Comparisons were made to determine effect by area and type of interface on seroprevalence. There was no significant difference in seroprevalence of *Anaplasma* spp. antibodies between Maun west and Chobe west (Table 2). Interestingly, seroprevalence of *B. bovis* in Maun west was significantly higher than in Chobe west (*p* = 0.0004) while *Babesia bigemina* was significantly higher in Chobe west compared to Maun west (*p* < 0.00001). Seroprevalence of *T. parva* in Maun west was more than twice that in Chobe west but not significantly different (Table 2). Mixed infections were found in this study. The results indicate that a total of 125 cattle out of 295 cattle had mixed infections. Cattle in Chobe west had a higher number of mixed infections compared to those in Maun west. The highest mixed infection consisted of *B. bigemina* and *Anaplasma* spp. at 27.8, 95% CI [23.0–33.2] followed by *B. bovis* and *Anaplasma* spp. at 8.5, 95% CI [5.81–12.2], *B. bigemina* and *B. bovis* at 2.5%, *B. bigemina* and *T. parva* at 0.7, 95% CI [0.19–2.44] *B. bovis* and *T. parva* at 0.7, 95% CI [0.19–2.44], respectively. Only one (1) animal in Maun west was infected by all the pathogens.

### A Comparison by Age and Sex

There was a significant difference between the ages of animals that harbored *A*. *marginale*. The infection was significantly higher in adults compared to calves (*p* = 0.0004). There was a significant association of the sex of animals with seroprevalence of *B*. *bovis*. The infection was significantly higher in females compared to males (*p* = 0.0004). Breed of cattle did not appear to predispose animals to the selected TBD pathogens although there was a tendency for *Anaplasma* infection to be slightly higher in indigenous Tswana cattle compared to other breeds (Table 2).

## Discussion

As it is the case for many sub-Saharan countries, there is a paucity of information on the occurrence and prevalence of ticks and TBDs of cattle in Botswana. The present survey was undertaken to determine the seroprevalence of selected TBDs pathogens, the abundance, diversity, and distribution of ticks infesting cattle at the wildlife-livestock interface in northern Botswana. Although, cross-sectional studies are limited at determining a cause-effect relationship, the findings of this study provide baseline data on ticks and TBDs pathogens that can inform the development of herd health management plan and guide future epidemiological studies. This study identified the presence of seven species of ticks: (*A. variegatum, R. evertsi*-*evertsi, R*. (*B*.) *decoloratus, R. appendiculatus, H. rufipes, H. truncatum*, and *R. sanguineus*). Tick species identified in our study (except for *R*. *simus* and *R*. *zambeziensis*) is in agreement with previous surveys implemented several decades ago in Botswana (25–27). Similarly, the distribution and composition of the identified tick species are in accordance with previous work (27) except for *R*. *sanguineous*. The presence of some tick species like *R*. *appendiculatus, R*. (*B*.) *decoloratus*, or *R*. *evertsi evertsi* in our study, were suspected based on findings of a previous study in buffalo populations from our study area (2). Similarly, the occurrence of outbreaks of *T. parva* in the area was suspected but never confirmed by clinical diagnosis. Overall, tick infestation was relatively high (73.4%). The observed tick burden among cattle in the study areas may be attributed to communal grazing practice that exposes animals to tick infested areas. This high tick infestation may increase the risk of occurance to tick borne pathogens. Strategic tick control during peak periods (summer) is necessary to allow ticks to naturally sustain endemic stability of TBDs through continuous challenge.

*Amblyomma variegatum, R*. *evertsi evertsi, R*. (*B*.) *decoloratus* were the most abundant and widely distributed tick species indicating high potential for transmission of heartwater, anaplasmosis, and babesiosis. *A*. *variegatum* was the most common tick in this study. This tick species is widely distributed through West, Central, East, and southern Africa including Zambia, north eastern Botswana, the Caprivi Strip of Namibia, northern part of Zimbabwe and central and northern Mozambique (17, 27). The observed prevalence of *A*. *variegatum* found in the current study is in agreement with Tesgera et al. (28), in Ethiopia who reported *A. variegatum* as the predominant tick species at a much higher prevalence (64%) than those found in our study. *A*. *variegatum* is an important vector of *Ehrlichia ruminantium*, the organism that causes heartwater in cattle (29). Moreover, this tick species is also incriminated in the epidemiology of *Dermatophilus congolensis* responsible for cutaneous streptothricosis that damages hides and skins of cattle (30). Owing to the prevalence of *A*. *variegatum* in this study, it would be relevant to explore the epidemiology of heartwater in the study sites.

*Riphicephalus evertsi evertsi* was the second most abundant and widely distributed species. This was not surprising considering that *R*. *evertsi evertsi* tolerates a wide range of climatic conditions (31) and has a widespread distribution, being the commonly found ixodid tick on livestock throughout most part of Africa (5) and in Botswana (27). Thus, it was not surprising that it was recorded in each sampled crush. Findings of the observed tick species is of great veterinary importance as these ticks are vectors of anaplasmosis (*R*. *evertsi evertsi*) and babesiosis (*Boophilus* spp.) found in this survey. Despite its low density, *R*. *appendiculatus* is the vector for the protozoan *T*. *parva*, the causative pathogen of East Coast Fever, Corridor Disease and Zimbabwean Theileriosis (17).

Tick species identified in this study such as *A*. *variegatum* and *H*. *rufipes marginatum* not only are of veterinary importance, but are vectors of zoonotic diseases such as Q fever, tick-bite fever, and Crimean–Congo haemorrhagic fever, respectively. The abundance of these tick species, may suggest that humans in the sampled sites are exposed to these diseases when bitten by infected ticks. Therefore, surveillance of TBDs in humans in the study areas is recommended.

Overall, we found higher tick infestations in the unfenced interface area compared to the fenced one, except for the case of *R. truncatum*. However, those variations of reported tick burdens were only significantly higher (*p* < 0.05) for *A*. *variegatum* and *R*. *evertsi evertsi*. This result may suggest an association of increased tick burden in cattle with a wildlife-livestock interface where there is no physical separation. However, in addition to the presence of physical barriers our results could have also been confounded by many other factors such as the complex interaction of several environmental and climatic conditions (annual rainfall, atmospheric temperature, and relative humidity), vegetation type, and host availability (32–34). Additionally, some tick species are ecological flexible and may easily adapt to changing climate and new habitats (35). The influence of these variables was not evaluated and may have influenced tick infestation and disease transmission. Moreover, climate change can change the geographical distribution of ticks and thus, influencing their occurrence, and pathogen transmission. Climatic variability is known to influence the tick habitat and the prevalence and incidence of haemoparasites (36). Seasonality also impacts tick infestation of livestock. Tick infestation in cattle is higher during the rainy season than the dry season since ticks have more successful reproduction cycles in wet and warm seasons than in dry cold periods (17, 37). One of the limitations of this study is that sampling was carried out in the winter season and this may have influenced tick population dynamics and infestation. Sampling in other seasons would have given a more accurate picture of the tick diversity in the study areas. Tick control methods may also influence the prevalence and endemic stability of TBDs in an area. In our study, information about acaricide treatment was not collected. However, we acknowledge that this could be another plausible reason for differences in the tick infestations between the study sites. This is an important limitation of our study and we recommend that future assessments of TBD's of this kind consider reporting and quantifying information on acaricide treatments and including these aspects in the interpretation of the results.

Small ruminants and wildlife are host to a diversity of tick species infesting cattle (38). Moreover, a study by Espinaze et al. (13), showed that small ruminants and dogs were important domestic animals for network connectivity with wildlife. This may facilitate cross-infestation with ticks and transmission of ticks and TB pathogens between wildlife and livestock at the interface areas (39). Therefore, we also recommend the inclusion of small stock other domestic animals, buffalo, and other wildlife animals in future investigations on the epidemiology and control of ticks and TB pathogens in future studies.

In this study, results of serological tests demonstrate the exposure of cattle to different vector borne pathogens. Considering that test performances of cELISA for *Anaplasma* spp. and IFAT for *Babesia* spp. and *Theileria parva* were quite high, results are fairly comparable. Antibodies to *Anaplasma* spp. was detected at a high frequency (90%) of the samples tested with cELISA. This finding is not surprising considering that *Anaplasma* has a wide vector range and can also be mechanically transmitted by biting flies (40, 41). In the south east region of Botswana, with comparatively less wildlife, Ramabu et al. (42) reported a similarly high prevalence of 91% *Anaplasma* spp. infection in beef and dairy cattle. This indicates that *Anaplasma* is widespread in the country and high prevalence cannot be associated with interaction of wildlife with cattle (41). Previous studies in eastern and southern African areas by Latif (43) and Dreyer et al. (44) have all indicated that *Anaplasma marginale* is widely distributed with prevalence rates ranging from 32.1 to 100%. Similarly, high prevalences of *Anaplasma* antibodies were reported in several studies in South Africa using ELISA (45, 46) with prevalences of 87, 98.2%, respectively. In contrast to our findings, lower seroprevalence of 32.1–40.0% were reported in several countries in East Africa such as Kenya (43, 47), Tanzania (48) and a much lower prevalence of 15% at wildlife-livestock interface areas in Uganda (49).

*Babesia begimina* was the main *Babesia* species found in the present study. The higher prevalence of *B*. *bigemina* compared to *B*. *bovis* may be explained by (i) wider vector range of *B*. *bigemina* (50), (ii) a higher concentration of the *B*. *bigemina* parasite in the capillary and veins than the *B*. *bovis* parasite which is evenly distributed in the whole blood vasculature (51, 52) (iii) tick infection rates are usually higher for *B*. *bigemina* (0.23%) than in *B*. *bovis* (0.04%) (53), with a consequent slower rate of transmission of the latter to cattle. Taken together, this would also suggest that, in an area where both species are present, endemic stability would be more likely to establish for *B*. *bigemina* compared to *B*. *bovis* (54). It is inconclusive whether physical separation between wildlife and cattle affected *Babesia* infection in cattle owing to the findings of the current study where *B*. *bigemina* infection was higher in Chobe west and *B*. *bovis* was higher in Maun west.

*Theileria parva* has been described in African buffalo in several locations in the region (55–59), including northern Botswana (2). The presence of *T. parva in* the buffalo population suggests that buffalo may serve as a reservoir hosts for either mechanical or biological transmission to cattle particularly where there is shared pasture and water at the wildlife-livestock interface. Thus, it was hypothesized that the prevalence of *T*. *parva* would be higher in Chobe west with a higher level of interaction between cattle and buffalo as described in South Africa (40). However, our findings do not support this hypothesis. The sample size was too small to warrant such a conclusion particularly given the low observed seroprevalence of 2.4%. The low seroprevalence could be due to a low infection challenge from ticks or that there are few ticks which could act as competent transmitters of the organism in the area. Furthermore, *T*. *parva* being transmitted transtadially; it is possible that infection was lost during the transmission process (60). Other possible explanations for the low seroprevalence may be that since the disease is fatal in cattle, it is difficult to find animals with antibodies that survived the infection. We recommend that confirmatory polymerase chain reaction (PCR) be carried out on seropositive samples similar to tests done on samples from buffalo by Eygelaar et al. (2).

The high *Anaplasma* infection prevalence suggests a situation of endemic stability of the pathogen in the cattle population. Clinical disease outbreaks in a population in which a pathogen has reached endemic stability are rare and clinical anaplasmosis is uncommon in both cattle populations investigated in the current study. *B. Bigemina, B*. *bovis*, and *T*. *parva* occur at prevalence level inconsistent with endemic stability and thus, animals in the population are susceptible to development of clinical disease. Cattle in Maun west and Chobe west are therefore, at risk of bovine babesiosis and theileriosis. It is worth-noting that although, endemic stability mitigates disease outbreaks, it is not reliable. Moreover, using seroprevalence alone does not give a clear indication of endemic stability status of TBDs in an area. Future studies with more data are necessary to make a comprehensive analysis of endemic stability of TBDs in the study sites. Additionally, it will be important to determine which strain of *Anaplasma* is endemic in the study sites and if possible, to detect tick borne pathogens determined in this survey by molecular methods directly in tick samples.

Overall, tick diversity and abundance observed in this study correlated with the seroprevalence of the selected tick borne pathogens.

## Conclusion

The results of this study provide the evidence of the important tick borne diseases pathogens and vectors responsible for their transmission in two important wildlife-livestock interface areas in northern Botswana. Our results suggest that irrespective of the type of interface (fenced or not fenced), ticks and TBD pathogens (*Anaplasma* spp., *B*. *bovis, B*. *bigemina*, and *T*. *parva*) are circulating in the two study areas with *Anaplasma* spp. being the most prevalent and *A*. *variegatum* and *R*. *evertsi evertsi* and *R*. (*B*.) *decolaratus* being the principal tick species infesting cattle in the study areas. Proper control measures against ticks and TBDs are necessary to improve cattle productivity and the livelihood of farmers in Maun west and Chobe west. Further epidemiological studies are needed to determine seasonal patterns of tick burdens and TBDs circulation in cattle and determine the economic impact of TBDs transmitted by the identified pathogens. Also to further clarify the relationship between livestock farming and wildlife conservation at the wildlife-livestock interface.

## Data Availability Statement

The datasets generated for this study are available on request to the corresponding author.

## Ethics Statement

Ethical review and approval was not required for the animal study because the sampling of cattle did not require any ethical approval. However, the project was discussed, approved by Department of Veterinary Services (Ministry of Agriculture), and undertaken with their direct collaboration in the field activities. Import permit was obtained from DVS for the movement of blood samples outside the foot and mouth disease infected area to targeted laboratories. Written informed consent was obtained from the owners for the participation of their animals in this study.

## Author Contributions

OR, SR, FJ, and LG designed the study. SR contributed cELISA reagents, testing, and analysis of test results. OR collected samples, identified ticks, and conducted serological test using cELISA. OR and FJ analyzed the data. LG assisted with production of maps. OR and SR prepared the first version of the manuscript. FJ and LG corrected and improved subsequent versions. All authors read and approved the final manuscript.

## Conflict of Interest

The authors declare that the research was conducted in the absence of any commercial or financial relationships that could be construed as a potential conflict of interest.
